# Real world evidence of epidemiological trends, clinical presentation, and prognostic outcomes of multiple myeloma (2007-2021)

**DOI:** 10.3389/fmed.2024.1338552

**Published:** 2024-02-20

**Authors:** Hesham Elsabah, Halima El Omri, Elmukhtar Habas, Ruba Y. Taha, Sarah A. ElKourashy, Feryal Ibrahim, Abdulqadir J. Nashwan, Nancy Kassem, Laxmi Ojha, Rajvir Singh, Rola Ghasoub, Abdelfatteh El Omri

**Affiliations:** ^1^Division of Hematology, Department of Medical Oncology, National Center for Cancer Care & Research (NCCCR), Hamad Medical Corporation, Doha, Qatar; ^2^Department of Internal Medicine, Hamad Medical Corporation, Doha, Qatar; ^3^Weill Cornell Medicine, Doha, Qatar; ^4^Department of Laboratory Medicine and Pathology, National Center for Cancer Care & Research (NCCCR), Hamad Medical Corporation, Doha, Qatar; ^5^Nursing Department, Hamad Medical Corporation, Doha, Qatar; ^6^Pharmacy Department, National Center for Cancer Care & Research (NCCCR), Hamad Medical Corporation, Doha, Qatar; ^7^Surgical Research Section, Department of Surgery, Hamad Medical Corporation, Doha, Qatar; ^8^Cardiology Research Department, Hamad Medical Corporation, Doha, Qatar

**Keywords:** multiple myeloma, survival rate, plasma cell, hypercalcemia, ASCT

## Abstract

**Background:**

Multiple myeloma (MM) is one of the most common hematological malignancies globally, and it is projected to increase in the coming years. It occurs more frequently in males and affects older individuals. Presenting symptoms can range from being asymptomatic to severely debilitating. The objective of this study was to determine the epidemiology, clinical features, and prognostic outcomes of patients with MM in the only tertiary cancer hospital in Qatar.

**Methods:**

Patients with symptomatic myeloma diagnosed at the National Center for Cancer Care and Research in Qatar between 2007 and 2021 were included. Data on demographics, laboratory work, bone marrow analysis, radiology, and given treatment were collected. Descriptive statistics, survival curves, and multivariable cox regression were used to identify independent mortality risk factors.

**Results:**

During the study period of 15 years, a total of 192 patients were diagnosed with MM. The incident rate of myeloma cases in 2021 was 8 patients per million. The median age of patients was 57 years [range 22–88], with 68% being above the age of 50 years at diagnosis. The majority of patients were male (71%) and (85%) were expats. At the time of diagnosis, most patients [*n* = 169 (88%)] had bone lesions, and 27% had extramedullary plasmacytoma. Anemia, hypercalcemia, and spinal cord compression were reported in 53%, 28%, and 7% of patients, respectively, at presentation. The monoclonal immunoglobulin subtypes were IgG, IgA, and free light chain in 52%, 16%, and 26% of patients, respectively. The overall median survival was 103 months (95% CI 71–135 months). In a multivariate cox-regression analysis for risk factors, only high serum calcium (≥ 2.7 mmol/L) was associated with increased mortality (HR: 2.54, 95% C.I.: 1.40–4.63, *p* = 0.002). Patients who received an autologous stem cell transplant (ASCT) had significantly better overall survival.

**Conclusion:**

In this comprehensive study of patients with MM treated in a country with a small and young general population, centralized hematology care, and free cancer care, we found a low but increasing incidence of MM and a good overall survival. Hypercalcemia was confirmed as a negative risk factor. ASCT had a significant positive impact on survival and should be provided to all patients eligible for this treatment, even in the era of novel agents.

## Introduction

Multiple myeloma (MM) is a neoplastic plasma cell disorder that accounts for 18% of hematological malignancies and 1.8% of cancer (1). The median age of patients at diagnosis is approximately 66–70 years, with 37% of patients being younger than 65 years old. The disease appeared to occur slightly more frequently in men. The incidence of MM is highly variable in different countries. However, the incidence of MM has consistently increased since 1990 (2). Worldwide, the incidence has increased by 126%, with 52.9% aging as a contributing factor. In the United States (USA), the lifetime risk of having MM is approximately 0.76% (2). The estimate for MM in the USA by the American Cancer Society for 2021 is approximately 35,000 new cases (19,320 men and 15,600 women). Furthermore, approximately 35% are expected to die (6,840 males and 5,570 females) per year (2). Australia and Western European countries have a higher age-standardized incidence and death rate, whereas in third-world countries, the population has a lower age-standardized MM incidence (3). Factors such as previous monoclonal gammopathy of undetermined significance (MGUS), occupation, and smoldering myeloma increase the risk of progression to MM (4). Furthermore, it was reported that distinct autosomal mutations in germline cells might prejudice MM (5). Although new therapies, such as proteosome inhibitors, immune modulatory agents, and monoclonal antibodies, are now available for MM treatment, the disease is still incurable, and morbidity and mortality rates are still high (2). This retrospective study is the most updated research, integrating significant information about the epidemiology, clinical manifestations, survival-related risk factors, and prognostic outcomes for patients with MM in the State of Qatar.

## Methods

A retrospective medical record review was conducted on 192 patients diagnosed with MM and treated at the National Center for Cancer Care and Research (NCCCR) in Qatar from January 2007 to December 2021. The NCCCR is the only hematology center in the country and provides free cancer care to all residents (a population of 2.6 million at the time of study end). Data were collected, verified, arranged in an Excel workbook, and then analyzed. The data included patients’ sex, age, nationality, laboratory workup, bone marrow (BM) aspiration and biopsy, and radiological features, among others. The results were presented as counts and percentages. The current study was approved by the Hamad Medical Corporation Medical Research Center Institutional Review Board (MRC-IRB) under the reference (MRC-01-17-104/01–21-986) for research entitled the clinicopathological profile of multiple myeloma in Qatar and was exempted from ethical approval.

### Statistical analysis

Descriptive statistics in the form of mean and standard deviation or median with IQR were presented for continuous variables age and duration, whereas categorical variables such as gender and ethnicity were presented in the form of frequency and percentages. Chi-square tests were used to examine the association between mortality and other independent categorical variables. Kaplan–Meier survival curves were used to assess median survival as well as other significantly associated factors using the chi-square test. A multivariable cox regression analysis was performed to identify independent risk factors for mortality after adjusting for age and sex. Hazard ratios and 95% C.I. were presented, and *p* values of 0.05 (two-tailed) were considered for statistically significant levels. The SPSS 28.0 statistical package was used for the analysis.

## Results

During the study period of 15 years, a total of 192 patients with a median age of 57 years (range 22–88) were diagnosed and treated for MM. Most patients [*n* = 131 (68.2%)] were above the age of 50 years, predominantly male (71%) and expats (84%). The clinical and diagnostic features of the included patients are summarized in Table 1. At presentation, [*n* = 46 (24%)] patients had hemoglobin (Hb) < 8 g/dL while [*n* = 55 (29%)] had Hb in the range of 8–10 g/dL. Hypercalcemia was found in [*n* = 52 (27%)] patients with serum calcium levels ≥2.7 mmol/L (normal range: 2.20–2.60 mmol/L). Renal impairment was found in [*n* = 67 (35%)] of patients with serum creatinine ≥133 mmol/L (normal range: 62–106 mmol/L) at diagnosis. Four patients with normal renal function had nephrotic range proteinuria, [*n* = 3 (1.5%)] patients had documented amyloidosis by kidney biopsy, and [*n* = 7 (3.6%)] patients required dialysis at diagnosis. Other rare presentations included bacterial infections in four patients and hyperviscosity syndrome in one patient.

**Table 1:** Description and association of socio-demographic and clinical characteristics with mortality.

Variable		Total, *n* = 192 (100%)	Alive, *n* = 128 (66.7%)	Dead, *n* = 64 (33.3%)	*p* value
Age (years)	≤ 50	61 (31.8)	41 (32)	20 (31.3)	0.91
	>50	131 (68.2)	87 (68)	44 (68.8)	
Gender	Male	137 (71.4)	94 (73.4)	43 (67.2)	0.37
	Female	55 (28.6)	34 (26.6)	21 (32.8)	
Ethnicity	Arabs	111 (57.8)	66 (51.6)	45 (70.3)	0.01
	Non-Arabs	81 (42.2)	62 (48.4)	19 (29.7)	
Nationals / residents	Qatari	31 (16.1)	16 (12.5)	15 (23.4)	0.05
	Non-Qatari	161 (83.9)	112 (87.5)	49 (76.6)	
Immunoglobulin types	IgG K&L	100 (52.1)	65 (50.8)	35 (54.7)	0.58
	IgA K&L	30 (15.6)	21 (16.4)	9 (14.1)	
	FLC K&L	50 (26.0)	34 (26.6)	16 (25.0)	
	Others*	12 (6.3)	8 (6.3)	4 (6.3)	
Secretory type immunoglobulin	Secretory	188 (97.9)	124 (96.9)	64 (100)	0.15
	Non-secretory	4 (2.1)	4 (3.1)	0 (0)	
Hemoglobin (g/dL)	<8	46 (24)	27 (21.1)	19 (29.7)	0.42
	[8, 10]	55 (28.6)	38 (29.7)	17 (26.6)	
	>10	91 (47.4)	63 (49.2)	28 (43.8)	
Creatinine (mmol/L)	<133	125 (65.1)	91 (71.1)	34 (35.1)	0.03
	[133, 177]	14 (7.3)	6 (4.7)	8 (12.5)	
	>177	53 (27.6)	31 (24.2)	22 (34.4)	
Total protein (g/L)	<60	9 (4.7)	5 (3.9)	4 (6.3)	0.19
	[60, 80]	68 (35.8)	51 (40.2)	17 (27)	
	>80	113 (59.5)	71 (55.9)	42 (66.7)	
Albumin (g/L)	<3.5	106 (55.2)	65 (50.8)	41 (65.1)	0.06
	≥3.5	85 (44.5)	63 (49.2)	22 (34.9)	
Calcium (mmol/L)	<2.7	138 (71.9)	99 (77.3)	39 (62.9)	0.04
	≥2.7	52 (27.4)	29 (22.7)	23 (37.1)	
Lactate Dehydrogenase	Elevated	28 (16.0)	16 (13.9)	12 (20.0)	0.30
	Normal	147 (84)	99 (86.1)	48 (80)	
Serum Beta-2 microglobulin (mg/L)	<5.5	103 (55.7)	75 (60.5)	28 (45.9)	0.06
	≥5.5	82 (44.3)	49 (39.5)	33 (54.1)	
International staging system	Stage I	38 (20.5)	28 (22.6)	10 (16.4)	0.17
	Stage II	65 (35.1)	47 (37.9)	18 (29.5)	
	Stage III	82 (44.3)	49 (39.5)	33 (54.1)	
Bone marrow infiltration	≥60%	94 (52.2)	63 (51.2)	31 (54.4)	0.69
	<60%	86 (47.8)	60 (48.8)	26 (45.6)	
Extramedullary involvement	No	135 (71.8)	93 (73.8)	42 (67.7)	0.75
	Para-Skeletal	40 (21.3)	25 (19.8)	15 (24.2)	
	Hematogenous	9 (4.8)	5 (4.0)	4 (6.5)	
	Mixed	4 (2.1)	3 (2.4)	1 (1.6)	
Upfront ASCT	Yes	81 (42)	68 (53.1)	14 (21.9)	0.001
	No	111 (58)	60 (46.9)	50 (78.1)	
Spinal cord compression	Yes	14 (7.4)	9 (7.0)	5 (8.2)	0.78
	No	175 (91.1)	119 (93.0)	56 (91.8)	
Follow up duration in months		42.3 ± 38.6	43.8 ± 39	39 ± 37	0.42

*N.B.: others include IgD, IgM, biclonal and non-secretory myeloma. ASCT: autologous stem cell transplant, hemoglobin (g/dL): normal range: 13–17, serum creatinine (mmol/L): normal range: 62–106, total protein (g/L): normal range: 60–80, calcium (mmol/L): normal range: 2.20–2.60, serum beta-2 microglobulin (mg/L): normal range: 0–3.

High plasma protein levels >80 g/L were observed in [*n* = 113 (59.5%)] of patients whereas plasma albumin <3.5 g/L was observed in [*n* = 106 (55%)]. On the other hand, serum beta-2 microglobulin ≥5.5 mg/L was significantly high in [*n* = 82 (44.3%)], and serum lactate dehydrogenase (LDH) was elevated in only [*n* = 28 (16%)] (Table 1).

Different radiological modalities evaluated the bone disease, with positron emission tomography (PET-CT) being the routine myeloma survey during the last few years; magnetic resonance imaging (MRI) spine was performed in 144 patients; X-ray skeletal survey in 68 patients; PET-CT scan in 91 patients; computed tomography (CT scan) in 17 patients; and it was not evaluated in 4 patients. Lytic bone lesions were detected in [*n* = 139 (72%)] patients, compression fractures in [*n* = 67 (35%)] patients, and pathological fractures in [*n* = 19 (10%)] patients, mainly involving ribs and femur bones. Extramedullary plasmacytoma was observed in [*n* = 53 (27%)] at diagnosis; para-skeletal, mainly paraspinal, is the most common site involved; other involved sites included the liver, skin, lymph nodes, pancreas, orbit, and bronchoalveolar. The symptom of spinal cord compression was the presenting myeloma manifestation in 7% of cases.

BM aspiration with plasma cell counts (<60%) and (≥60%) were observed in [*n* = 86 (48%)] of patients and [*n* = 94 (52%)] patients, respectively (Table 1). Regarding the prognostic MM International Staging System (ISS), stage III was the most common at diagnosis in [*n* = 82 (44.5%)] of patients, followed by stage II [*n* = 65 (35%)] and stage I in [*n* = 38 (20.5%)] of patients. IgG myeloma was reported in 52%, IgA myeloma in 16% of patients, free light chain myeloma in 26%, and others in 6%. Fluorescence *in situ* hybridization (FISH) analysis on interphase nuclei using IGH, TP53/CEP17, RB1/CEP10, and IGH/CCND1 probes and conventional metaphase karyotyping on bone marrow specimens was available for 149 patients and revealed 13q abnormalities (deletion/monosomy) in [*n* = 24 (16%)], hyperdiploidy in [*n* = 12 (8%)], *t*(11;14) in [*n* = 11 (7.3%)], complex karyotype in [*n* = 11 (7.3%)], trisomy 11 in [*n* = 5 (3.3%)], 17p deletion in [*n* = 5 (3.3%)], and hypoploidy in [*n* = 3 (2%)]. Monosomy 14, 1p deletion, *t*(14:16), and *t*(X:14) were found in one patient each.

The choice of first-line treatment evolved together with the available scientific evidence, with the majority of patients receiving the VCD (bortezomib, cyclophosphamide, and dexamethasone) regimen shifting to the VRD (bortezomib, lenalidomide, and dexamethasone) regimen and then to VRD-Daratumumab through the study period. The treatment protocols used during the study period are summarized in Table 2. A total of [*n* = 90 (47%)] underwent stem cell transplantation during the disease course. Seventy-seven patients had a single autologous stem cell transplant (ASCT), [68 ASCT upfront line therapy, 9 ASCT undergone during disease relapse], 5 tandem ASCT, and 8 double ASCT during their disease course. 52 patients had ASCT in our center and 29 patients in abroad centers. The maintenance therapies post the upfront ASCT were lenalidomide [*n* = 61], thalidomide [*n* = 4], pomalidomide [*n* = 1], carfilzomib [*n* = 1], VRD [*n* = 1], and no maintenance [*n* = 13]. Follow-up assessment showed that one patient developed secondary AML and five patients progressed to plasma cell leukemia.

**Table 2:** MM patients first line therapy during 2007–2013 and 2014–2021.

Therapy		Patients	2007–2013	2014–2021
Proteasome inhibitors based therapy	VCD [cyclophosphamide-dexamethasone]	65	15	50
	VRD [lenalidomide—dexamethasone]	41	_	41
	PAD [doxorubicin—dexamethasone]	14	14	_
	VTD [thalidomide-dexamethasone]	2	2	_
	VD [dexamethasone]	5	1	4
	D-VRD [daratumumab–lenalidomide-dexamethasone]	19	_	19
	VMP [melphalan-dexamethasone]	2	2	-
	D-VCD [daratumumab-cyclophosphamide-dexamethasone]	2	-	2
V: Bortezomib				
Immunomodulatory (IMIDs) Therapy	LD [lenalidomide –dexamethasone]	7	2	5
	CTD [thalidomide –cyclophosphamide-dexamethasone]	3	2	1
	TD [thalidomide -dexamethasone]	3	2	1
	T-DOX [thalidomide –doxorubicin]	2	2	_
	MPT [thalidomide-prednisone-melphalan]	3	3	_
	DRD [daratumumab-lenalidomide-dexamethasone]	1	_	1
Other	Hyper-CVAD [vincristine-dexamethasone- doxorubicin – cyclophosphamide]	1	_	1
	MP [prednisone-melphalan]	1	1	_
	Dexamethasone	3	3	_
	VAD [vincristine-dexamethasone- doxorubicin]	2	2	_
	N/A [not available]	16	10	6
Total number of patients		192	61	131

The overall response rate (ORR) to first-line therapy was 78%, with 39% of patients having a complete response (CR), 21% having a very good partial response (VGPR), and 18% having a partial response (PR), while refractory and progressive disease was observed in 8% of patients. The non-evaluated patients, due to a lack of data, represented 14% of the population study (Table 3).

**Table 3:** Distribution of response rate to first-line therapy.

Criteria	Details	*n* (%)
Response rate to first line therapy	ORR	150 (78.1)
	CR	75 (39)
	VGPR	40 (20.8)
	PR	35 (18.2)
	Refractory/Progression	15 (7.8)
	N/A	27 (14)

ORR, overall response rate, CR, complete response, VGPR, very good partial response, PR, partial response, N/A, not available.

**Figure 1:**
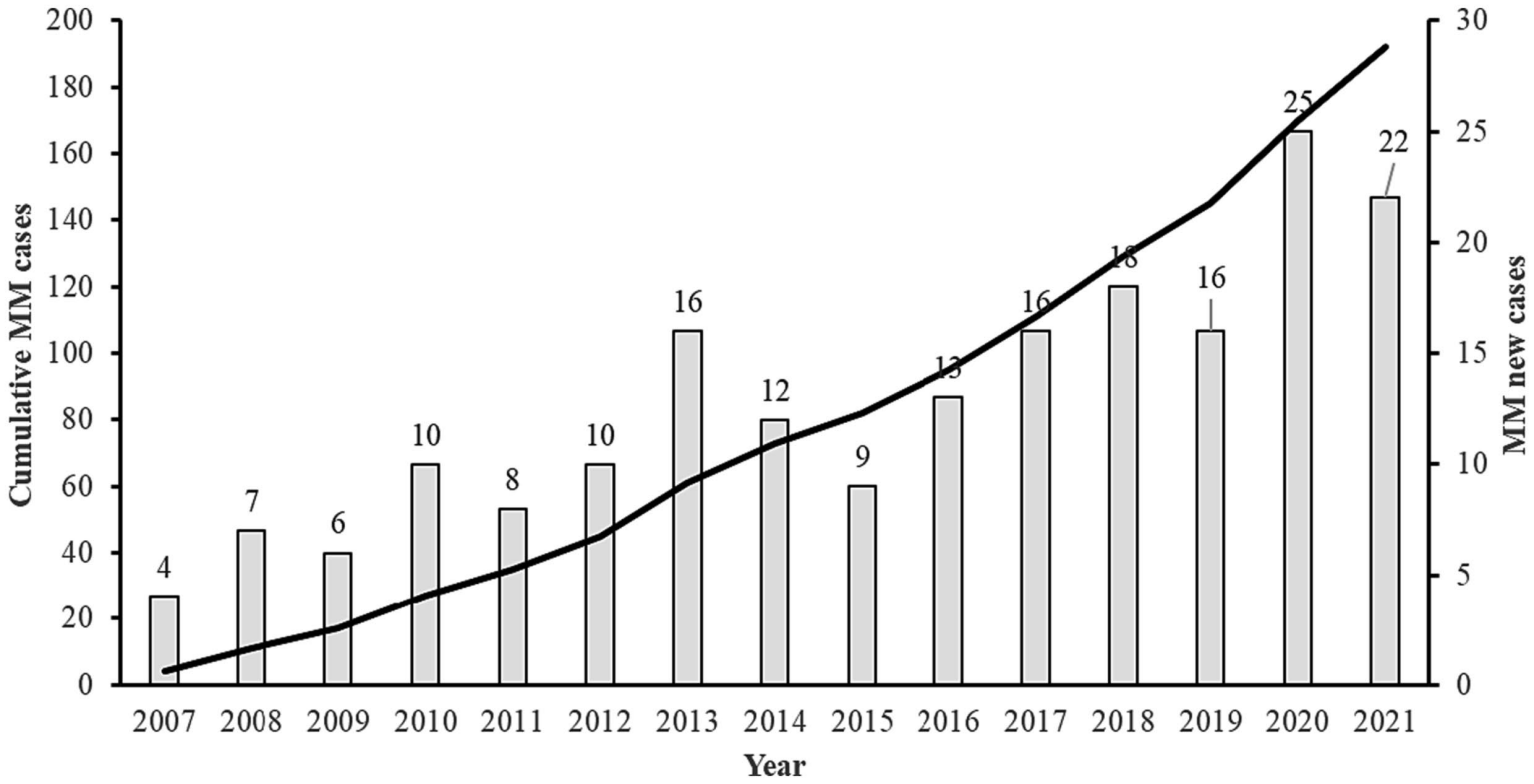
Annual distribution of multiple myeloma in the state of Qatar including MM new cases and cumulative cases from 2007 to 2021.

In our investigation, we observed fluctuations in the annual count of newly diagnosed patients across distinct time intervals. Notably, the average number of newly diagnosed patients per year was 7 from 2007 to 2011, increased to 12 patients annually during the period from 2012 to 2016, and further rose to 19 patients per year from 2017 to 2021 (Figure 1).

**Figure 2:**
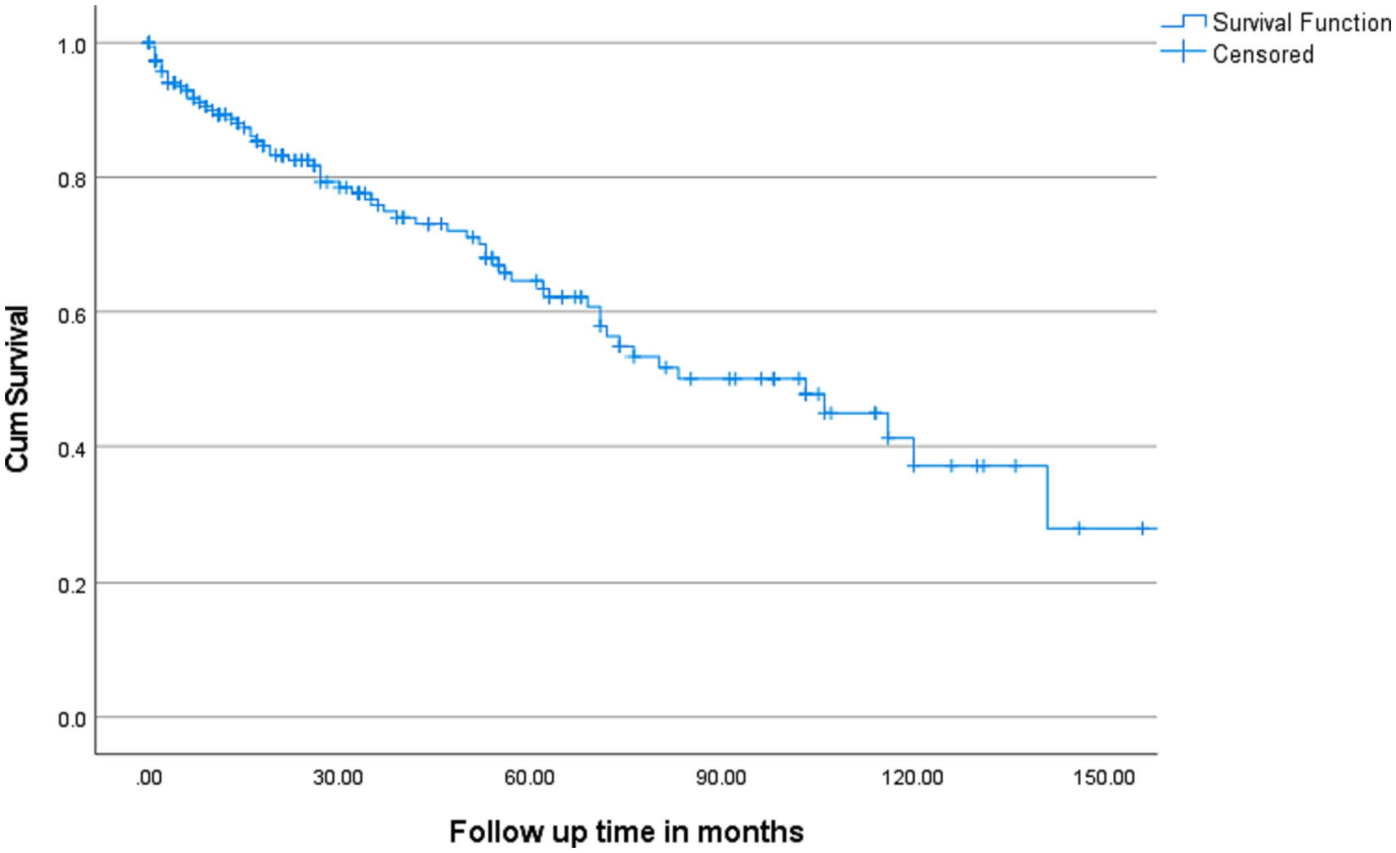
Kaplan–Meier estimates of Overall Survival (OS) in patients with MM. Overall median survival was 103 months with 95% C.I. (71–135 months).

**Figure 3:**
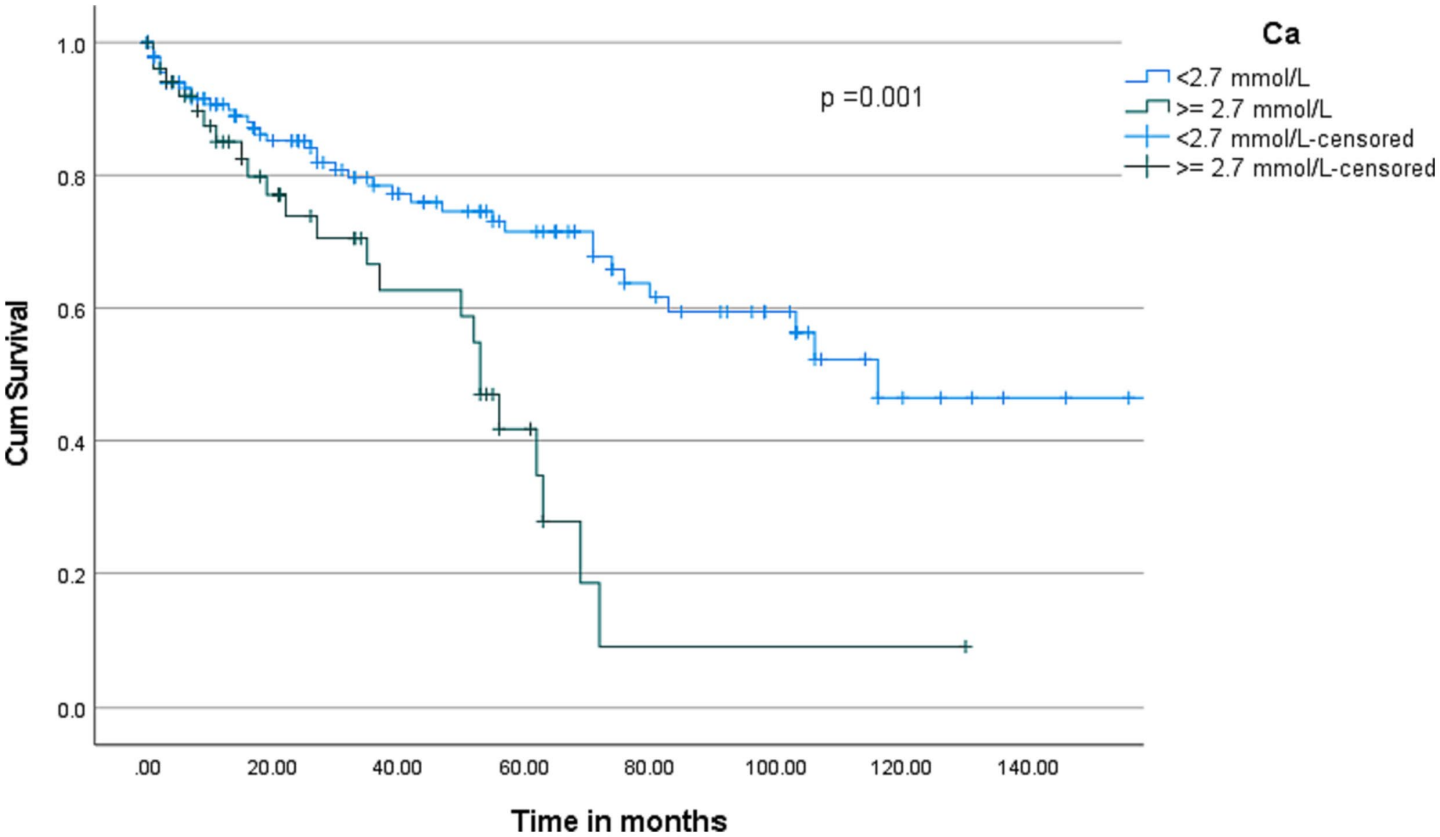
Kaplan–Meier curves of overall survival according to serum calcium levels. Compared with patients with serum calcium levels ≥ 2.7 mmol/L, death was significantly lower in patients with serum calcium levels < 2.7 mmol/L (*p* < 0.001).

**Figure 4:**
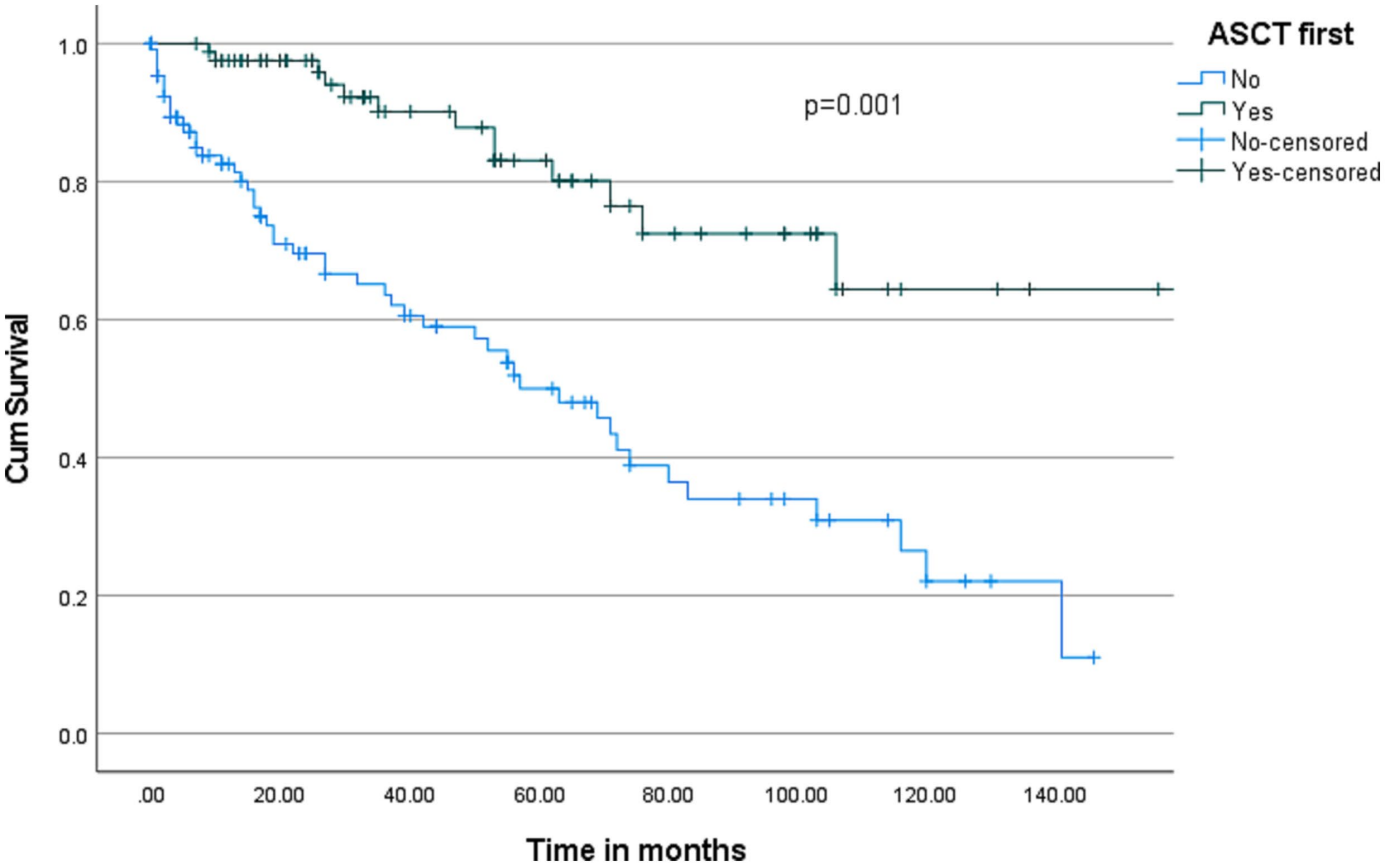
Kaplan–Meier survival curve for patients who received and who did not receive ASCT (Autologous Stem Cell Transplantation).

The overall median survival was 103 months with a 95% C.I. of 71–135 months (Figure 2). Multivariate cox-regression analysis showed that serum calcium ≥2.7 was associated with higher mortality in MM patients (HR: 2.55, 95% C.I.: 1.40–4.63, *p* = 0.002) after adjusting other independent variables such as age, sex, ethnicity, and creatinine (Table 4). To evaluate the prognostic value of the serum calcium level in MM patients followed up for 150 months, patients were categorized into two groups according to serum calcium level (2.7 mmol/L). Deaths were [*n* = 39/138 (28.3%)] in the <2.7 mmol/L group and [*n* = 23/52 (44.2%)] in the serum calcium level ≥ 2.7 mmol/L group (*p* < 0.001). Our plot shows that patients with a serum calcium level < 2.7 mmol/L have a higher probability of survival almost throughout the whole time period (Figure 3). The OS of MM patients who underwent ASCT was significantly better over time compared to patients without ASCT (*p* < 0.0001). Kaplan–Meier estimates showed that mortality was lower in patients who underwent ASCT (Figure 4).

**Table 4:** Factors associated with mortality: a multivariate cox regression analysis.

Variable	Adjusted HR ratio	95% C.I.	*p* value
Age (≥50 years)	0.88	0.50–1.54	0.65
Gender (Male)	0.90	0.51–1.60	0.72
Arab	1.20	0.68–2.10	0.56
Serum creatinine (mmol/L)^*^			
<133	1	Reference	
133–177	2.93	1.30–6.68	0.01
>177	1.60	0.88–2.87	0.12
Calcium ≥2.7 mmol/L^**^	2.54	1.40–4.63	0.002

* normal range: 62–106 mmol/L, ** normal range: 2.20–2.60 mmol/L.

## Discussion

This is the first comprehensive study of patients with MM in a country with a small and young general population, centralized hematology care, and free cancer care. The MM burden is anticipated to expand across the world’s countries. The worldwide yearly incidence of MM is estimated to be 160,000, and the global myeloma mortality amounted to 106,000 patients in 2018 (6). This translates into a global age-standardized incidence and mortality rate of 2.1 per 100,000 and 1.39 per 100,000, respectively (6). With an incidence of 0.8, Qatar belongs to the populations with a very low MM incidence. This probably reflects the very young age of our general population compared with the USA and Europe. According to the Planning and Statistics Authority in Qatar, the population of the country was about 2,677,001 in December 2021, and it was predominantly urban, with almost 85% being expats. The median age of the population is estimated to be 32–33 years (7).

The average increase in newly diagnosed MM cases in Qatar from 2007 to 2021 was 13 ± 6 patients per year, with a significant increase since 2016. In 2020, the total number of new cancer patients in Qatar was 1,482. Approximately 15% of them had breast cancer, leukemia (6.3%), non-Hodgkin lymphoma (5.9%), and MM (1.3%) (8). The number of newly diagnosed MM cases in Qatar has increased significantly, from 4.5 per million in 2008 to 8.2 per million in 2021. Although the number of newly diagnosed cases doubled in 15 years, Qatar still shows the same or a lower incidence of MM than other parts of the world (6, 9, 10). This increase could be due to the rise in the elderly population and the late improvement in clinical care and diagnosis.

MM is a disease of the elderly and is more common in men than in women (≈1.4:1), especially in African American descendants. The reported median age of MM diagnosis is between 65 and 74 years, although MM was reported in 10 and 2% of patients <50 and < 40 years old (11, 12). The present study showed that the median age of the affected population was 57 years, indicating that MM affects the Qatari population at a young age. This most probably reflects the selection caused by the young general population, as discussed above. MM incidence also varies by ethnicity, and it was reported that Asians had a relatively lower incidence than Caucasians (13), although another Korean study in 2010 suggested an increase in MM incidence (14). In the current study, MM was observed in Arabs (58%), including 16% Qatari nationals. Since the population of Qatar is extremely multi-ethnic, no conclusion can be drawn.

Although anemia can improve with chemotherapy, it affects 60 to 70% of MM patients either at the diagnosis or during the disease course (11, 15–18). In our population, anemia (Hb ≤ 10 gm/dl) was observed in 53% of patients, agreeing with previous studies (10–12, 16, 19). Anemia in MM patients is mainly due to malignant plasma cells and bone marrow infiltration, which affects red blood cell production and survival (20). Furthermore, MM causes renal impairment, which can precipitate anemia (21).

As it is well known in MM, anemia in our patient cohort can be explained by the presence of renal impairment (confirmed in 35% of the patients) and a heavy infiltration of BM by the plasma cells (confirmed in 50% of patients).

Bone diseases, including lytic lesions, vertebral compression, and pathological fractures, were observed in almost 90% of cases at presentation, and extramedullary involvement was observed in 27% of cases. Bone disease is the main cause of morbidity and disability in MM, and its incidence in our population study is almost similar to reported data (22) while extramedullary disease (EMD) involvement is slightly higher than that in other studies (23). The more liberal use of sensitive diagnostic modalities such as PET-CT and MRI may explain the higher rate of EMD involvement in our patients at diagnosis.

MM is classified into two types: secretory and non-secretory. The secretory type includes IgG, IgA, IgE, IgM, and light-chain MM (24). In our study, IgG myeloma was the most common subtype, which is consistent with previous studies (24). Our study reported a higher incidence of light chain myeloma in the included patients, with no significant impact on survival, as the majority of these patients were treated with bortezomib-based therapy, which is highly effective in treating this subgroup of MM.

Cytogenetic abnormalities are observed in most MM patients. The prognostic value has been thoroughly investigated, with limited data on the relationship between primary cytogenetic abnormalities and disease characteristics and the response to available treatments. Abdallah et al. reported an association between high-risk disease characteristics and immunoglobulin heavy chain (IgH) translocations and trisomies, leading to better responses to proteasome inhibitors and immunomodulatory drugs (IMiDs) (25).

The most common cytogenetic abnormalities observed in our patients were 13q abnormalities, followed by hyperdiploidy and complex karyotypes. The frequency of the genetic aberrations might have been underestimated since the cytogenetic analysis was done on marrow specimens and not on CD138-positive-enriched samples, as the CD138 selection technique had not yet been put into practice.

MM is a treatable, but not curable, disease. The survival rate of MM has improved significantly in recent years, with a 5-year survival rate now around 54% (26) and varying depending on the stage of the disease, treatment availability, response to treatment, and other individual risk factors. In the current study, we found that the overall median overall survival was 8.5 years with 95% confidence interval (C.I. 71–135 months).

Analysis of risk factors in relation to mortality in our patient cohort revealed hypercalcemia as the only negative prognostic factor. Hypercalcemia is a common feature of MM. It is of multifactorial etiology in these patients and is known to be associated with a poor prognosis (27). The management of this disease complication is usually integrated into the urgent treatment of newly diagnosed MM. If it is not managed properly, it may lead to more serious complications, such as kidney damage, cardiac arrhythmia, and coma (27). Therefore, regular monitoring of serum calcium levels and bone health in MM is crucial for monitoring disease progression and designing an appropriate treatment plan.

Our study confirms the positive role of ASCT in the management of patients with MM eligible for intensive chemotherapy.

## Conclusion

In this comprehensive study of patients with MM treated in a country with a small and young general population, centralized hematology care and free cancer care, we found a low but increasing incidence of MM and a good overall survival. Hypercalcemia was confirmed as a negative risk-factor. ASCT had a significant positive impact on survival and should be provided to all patients eligible for this treatment even in the era of novel agents.

## Data availability statement

The original contributions presented in the study are included in the article/supplementary material, further inquiries can be directed to the corresponding author.

## Author contributions

HE: Writing – original draft. HaE: Methodology, Writing – original draft. EH: Writing – review & editing, Validation. RT: Writing – review & editing. SE: Methodology, Writing – review & editing. FI: Writing – review & editing. AN: Writing – review & editing. NK: Writing – review & editing. LO: Writing – review & editing, Data curation. RS: Writing – review & editing. RG: Validation, Writing – review & editing. AE: Writing – review & editing.
